# Atomic scale insight into the decomposition of nanocrystalline zinc hydroxynitrate toward ZnO using Mn^2+^ paramagnetic probes

**DOI:** 10.3389/fchem.2023.1154219

**Published:** 2023-04-06

**Authors:** Ioana D. Vlaicu, Mariana Stefan, Cristian Radu, Daniela C. Culita, Dana Radu, Daniela Ghica

**Affiliations:** ^1^ National Institute of Materials Physics, Magurele, Romania; ^2^ Faculty of Physics, University of Bucharest, Magurele, Romania; ^3^ Ilie Murgulescu Institute of Physical Chemistry, Romanian Academy, Bucharest, Romania

**Keywords:** zinc hydroxynitrate, nanocrystals, electron paramagnetic resonance, Mn^2+^ paramagnetic probe, morpho-structural analysis, structural phase transformation

## Abstract

Layered zinc hydroxynitrate (ZHN), with the chemical formula Zn_5_ (OH)_8_ (NO_3_)_2_·2H_2_O, exhibits a range of special properties such as anion-exchange and intercalation capacity, as well as biocompatibility, making it attractive for a large variety of applications in fields from nanotechnology to healthcare and agriculture. In this study nanocrystalline ZHN doped with 1,000 ppm Mn^2+^ was prepared by two synthesis methods (coprecipitation and solid state reaction) using similar environment-friendly precursors. The complex morpho-structural [X-ray diffraction, scanning and transmission electron microscopy, textural analysis] and spectroscopic [Fourier transform infrared and electron paramagnetic resonance (EPR)] characterization of the two ZHN nanopowders showed similar crystalline structures with Mn^2+^ ions localized in the nanocrystals volume, but with differences in their morphological and textural characteristics, as well as in the doping efficiency. ZHN obtained by coprecipitation consists of larger nanoplatelets with more than two times larger specific surface area and pore volume, as well as a dopant concentration than in the ZHN sample obtained by solid state reaction. The thermal stability and the on-set of the structural phase transformation have been investigated at atomic scale with high accuracy by EPR, using Mn^2+^ as paramagnetic probes. The on-set of the ZHN structural phase transformation toward ZnO was observed by EPR to take place at 110°C and 130°C for the samples prepared by coprecipitation and solid state reaction, respectively, evidencing a manganese induced local decrease of the transformation temperature. Our results contribute to the selection of the most appropriate ZHN synthesis method for specific applications and in the development of new green, cost-effective synthesis routes for Mn^2+^ doped nano-ZnO.

## 1 Introduction

After the discovery of polymeric nanocomposite materials containing mineral clays, a considerable increase in the scientific interest for layered compounds was recorded, especially for the layered double hydroxide salts (LDHS) (Braterman et al., 2004; Oh et al., 2009). Meanwhile, the structurally similar layered hydroxide salts (LHS) exhibit the same interesting properties such as anion-exchange and intercalation capacity, which enable their use as catalysts and matrices of functional nanocomposites with polymers. (Newman and Jones, 1999; Wypych et al., 2005; Biswick et al., 2006; Marangoni et al., 2009; Cursino et al., 2010; Machado et al., 2010; Reinoso et al., 2014; de Oliveira and Wypych, 2016; Ghotbi et al., 2018). Other applications in nanomedicine, agriculture and cosmetics are related to the biocompatibility of these layered materials, e.g., slow releasing drug delivery agents, biomolecule reservoirs, fertilizers, herbicides, sunscreens (Oh et al., 2009; Cursino et al., 2010; Li et al., 2012; Ruiz et al., 2020).

A typical representative of LHS is zinc hydroxynitrate, with molecular formula Zn_5_ (OH)_8_ (NO_3_)_2_·2H_2_O (ZHN). One of the problems limiting the large scale use of this compound for most of these applications is its thermal stability. The thermal stability of ZHN was investigated in the past five decades by several groups and several different mechanisms for the thermal decomposition to zinc oxide (ZnO) were proposed. However, most of the studies report the same two overlapping steps for the decomposition, below 200°C or between 200°C and 320°C, depending on the synthesis or post-synthesis conditions, steps that involve the formation of intermediates like anhydrous Zn_5_ (OH)_8_ (NO_3_)_2_ and Zn_3_ (OH)_4_ (NO_3_)_2_ (Stählin and Oswald, 1971; Biswick et al., 2007; Ahmadi et al., 2013).

It is expected that the morpho-structural properties affect material’s efficiency for specific applications. Therefore, it is important to establish performant and reproducible synthesis algorithms, resulting in materials with defined morpho-structural characteristics, in view of a larger scale use of ZHN. To our knowledge, there are few reports containing information on the morphology of the investigated ZHN (Hussein et al., 2009; Li et al., 2012; Reinoso et al., 2014; de Oliveira and Wypych, 2016; Ruiz et al., 2020), while the ZHN crystallinity degree and crystallite size are mentioned only by Hussein et al. (2009). No discussion is available in literature on the influence of the ZHN morpho-structural aspects on the material thermal stability and its structural phase transformation path.

Electron paramagnetic resonance (EPR) spectroscopy of lightly doped (nano)-crystals has been successfully used to investigate structural phase transformations at atomic scale using paramagnetic probing ions, resulting in a richer information at atomic level and higher accuracy than in the case of thermal analysis methods (Nistor et al., 2014). Mn^2+^ proved to be a good paramagnetic probe in zinc based compounds, due to the similar charge state and close ionic radii with the host Zn^2+^ ions (Nistor et al., 2013; Stefan et al., 2013; Ghica et al., 2014). In low concentrations (below 1% nominal concentration), the Mn^2+^ doping ions do not affect the host lattice and allow the determination with high sensitivity and accuracy of the on-set of the phase transformations (Ghica et al., 2011; Nistor et al., 2013; Ghica et al., 2019). Moreover, due to the EPR sensitivity which is much higher than that of the X-ray diffraction (XRD) technique, low concentrations of doped minority phases could be identified as well (Ghica et al., 2011; Nistor et al., 2013; Ghica et al., 2016).

In answer to all aspects and problems discussed above, we have synthesized nanostructured ZHN by two cost-effective methods, using the same environment-friendly precursors and investigated the morpho-structural properties and thermal stability of the resulting Mn-doped ZHN nanostructured powders, within an original approach. The localization of the low concentration Mn^2+^ ions and the doping efficiency in the two ZHN nano-systems, of particular interest for applications, were evaluated by EPR. The structural phase transformation was monitored at atomic scale by EPR with the Mn^2+^ probing ions, showing with high accuracy the on-set of the structural transformation toward ZnO and the differences in the two nano-systems.

The importance of this work is better evidenced in a larger context, as it opens the way to applications in a wide range of fields: i) green, cost-effective ZHN synthesis by different methods, for applications from nanotechnology to healthcare and agriculture; ii) developing new cost-effective synthesis routes for Mn^2+^ doped nano-ZnO. ZnO is one of the most intensively studied semiconductor with already a wide range of applications from nano- and/or optoelectronics to multiple bio-medical applications (Fortunato et al., 2009; Hoye et al., 2013; Verma et al., 2021). Furthermore, by doping it is possible to tailor or develop new material properties, extending the range of applications (Sharma et al., 2003; Dietl, 2010; Zhang et al., 2018; Pradhan, 2019; Popescu et al., 2020; Pradeep and Viswanatha, 2020).

## 2 Materials and methods

### 2.1 Materials synthesis

Zn_5_ (OH)_8_ (NO_3_)_2_·2H_2_O has been synthesized by two methods, using the same precursors without any further purification: zinc nitrate hexahydrate [Zn (NO_3_)_2_·6H_2_O, from Sigma Aldrich], manganese nitrate tetrahydrate [Mn (NO_3_)_2_·4H_2_O, from Alfa Aesar] and sodium hydroxide [NaOH, from Sigma Aldrich]. The preparation procedures for the two materials, labeled ZHN_COPP_ and ZHN_SSR_ after the coprecipitation and solid state reaction methods used, are detailed below:

ZHN_COPP_: Coprecipitation method—an aqueous solution containing zinc nitrate and manganese nitrate, in the corresponding amounts for 1,000 ppm Mn^2+^ nominal concentration, was slightly acidified with 250 mL HNO_3_ (1 M) and magnetically stirred for several minutes. Afterwards, this mixture was precipitated with a strong basic solution of NaOH 2.4 M (excess was used) added slowly with a peristaltic pump, at nearly 35°C, under continuous magnetic stirring. The suspension thus obtained was allowed to age for 1 hour at the same temperature, under continuous magnetic stirring. The precipitate was collected by centrifugation and washed several times with bi-distilled water and absolute ethanol, and air dried at 60°C.

ZHN_SSR_: Solid state reaction method—zinc nitrate hexahydrate, manganese nitrate tetrahydrate (the amount corresponding to a 1,000 ppm Mn^2+^ nominal concentration) and sodium hydroxide were thoroughly mixed and ground in an agate mortar, in a molar ratio 1:0.62, without any water or other solvents added, until a homogenous white paste was formed. The paste was collected in a beaker and was carefully washed with bi-distilled water several times. The white precipitate separated by centrifugation was air dried at 60°C.

### 2.2 Materials characterization

XRD patterns were recorded with a Bruker D8 Advance X-ray diffractometer with Cu anode and Ni filter (λ = 0.154184 nm), in Bragg-Brentano geometry. The structural parameters of the identified crystalline phases were determined by Rietveld refinement of the experimental XRD data with the Bruker Topas v. 3 software.

Morpho-structural investigations down to the nano-scale were performed by scanning electron microscopy (SEM) and transmission electron microscopy (TEM) using Tescan Lyra III FEG and JEM 2100 electron microscopes, respectively.

Nitrogen adsorption-desorption isotherms at −196°C were recorded on a Micromeritics ASAP 2020 apparatus. Both samples were degassed at 70°C for 15 h, under vacuum, prior to N_2_ adsorption. The analysis of the isotherms allowed us to determine the specific surface area (S_BET_), according to the Brunauer–Emmett–Teller (BET) equation, the total pore volume (V_total_) from the amount adsorbed at the relative pressure of 0.995, and the mean pore diameter using Barrett–Joyner–Halenda (BJH) method.

Fourier transform infrared (FTIR) spectra were recorded with a Spectrum BX II (Perkin Elmer) spectrometer in absorbance mode, in the 4,000 cm^−1^–400 cm^−1^ spectral range, with 32 scans and a resolution of 4 cm^−1^. The samples were embedded in KBr pellets with a mass-ratio sample: KBr of 1:100.

EPR investigations were performed on weighted amounts of powders inserted in calibrated pure fused silica sample tubes of 2 mm inner diameter. X (9.8 GHz) and Q (34 GHz) -band EPR measurements were performed at room temperature (RT) with the Bruker ELEXSYS E580 and E500 spectrometers, respectively. The magnetic field calibration at the sample was made with a BDPA (alpha, gamma–bisdiphenylene-beta-phenyl allyl) reference sample from Bruker, exhibiting a single EPR line with g = 2.00276. The spin Hamiltonian (SH) parameters of the observed paramagnetic centers were determined with the EASYSPIN v.5.2.28 software (Stoll and Schweiger, 2006). Isochronal annealing experiments from 100°C up to 150°C were performed in air in steps of 10°C for 10 min at each temperature in order to study the thermal evolution of the samples inside the EPR tubes. The samples were sequentially annealed at successive temperatures in a temperature stabilized (±1°C) furnace and cooled down to RT for further measurements. Small amounts of samples extracted from the EPR tube after specific annealing treatments were deposited on a zero-diffraction Si wafer (MTI Corporation, USA) for XRD measurements.

## 3 Results and discussion

### 3.1 XRD investigation

**FIGURE 1:**
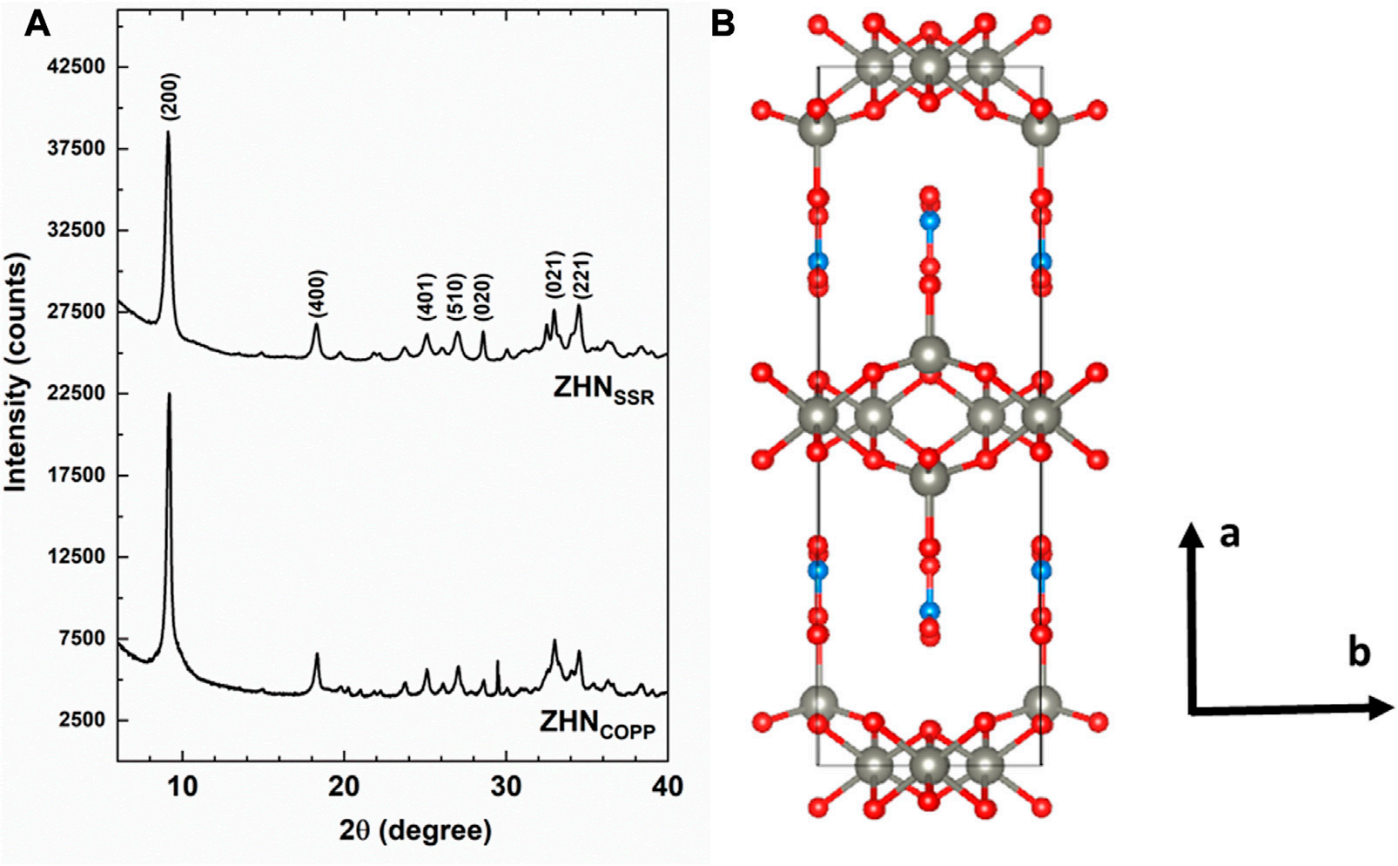
**(A)** XRD patterns of the as-prepared ZHN samples. Only the most intense peaks of the Zn_5_ (OH)_8_ (NO_3_)_2_ (H_2_O)_2_ structure have been marked. **(B)** Structural model along the *c-axis* with the following color codes for atoms: grey–Zn, red–oxygen, light blue–nitrogen.

Both ZHN_COPP_ and ZHN_SSR_ samples (Figure 1A) exhibit X-ray diffractograms indexed as pure zinc hydroxynitrate hydrate–Zn_5_ (OH)_8_ (NO_3_)_2_·2H_2_O, monoclinic structure, space group C2/m, ICDD 04-011-5271. The Rietveld refinement of the experimental data resulted in similar structural parameters: *a* = 1.9480 nm, *b* = 0.6238 nm, *c* = 0.5517 nm, *α* = 90^o^, *β* = 93.28^o^, *γ* = 90^o^, and average crystallite size *d* = 25 nm ± 3 nm. A pronounced (200) texture is observed in the case of the ZHN_COPP_ sample, with a corresponding coherence distance on the [200] direction of 40 nm ± 3 nm. As the XRD texture could be associated with the shape anisotropy of the nanocrystals, this aspect has been investigated by electron microscopy in Section 3.2.

Figure 1B presents a view along the *c*-axis of the structural model of the ZHN compound with the structural parameters from above. According to previous structural investigations (Stählin and Oswald, 1970; Stählin and Oswald, 1971; Chouillet et al., 2004; Biswick et al., 2007), the layered structure of Zn_5_ (OH)_8_ (NO_3_)_2_·2H_2_O consists of Zn (OH)_2_ layers with CdI_2_ type structure. In each such layer one-quarter of the zinc atoms are missing from the octahedral sites, while each occupied octahedron shares its edges with four occupied and two unoccupied octahedral sites. Tetrahedrally coordinated Zn^2+^ cations are located above and below the empty octahedra, coordinated in three corners of the tetrahedron by hydroxide ions from the brucite-like layers and in the fourth corner by a water molecule. The nitrate anions are not directly coordinated to the zinc ions, being located between the sheets and lying in a plane normal to them, preserving the *D*_*3h*_ symmetry. The hydrogen bonds holding together the sheets bind the oxygen atoms of the nitrate groups with the water molecule and with two hydroxide ions of the sheet.

It should be mentioned that the presence of the Mn^2+^ ions with 0.1% nominal concentration in the host ZHN lattice would not be observable by XRD investigations. The Mn^2+^ ions have similar charge state and close ionic radii with the host Zn^2+^ ions (Shannon, 1976). The substitution of the tetrahedrally coordinated Zn^2+^ ions (crystal radius R = 0.074 nm) by the slightly larger Mn^2+^ ions (R = 0.080 nm) would produce a small expansion of the host lattice associated with lattice disorder. This would be evidenced by an increase of the lattice parameters and a decrease of the crystallite size, observed in XRD patterns as shifts in the peak positions and line broadening, respectively. However, at low dopant concentration this effect is reduced, and below 1% nominal concentration the Mn^2+^ doping ions would not affect the host lattice in a detectable way (Ghica et al., 2011; Nistor et al., 2013; Ghica et al., 2019), as confirmed by our XRD results. Instead, the localization of the Mn^2+^ ions and the annealing induced changes in their neighborhood/environment were investigated by the more sensitive EPR spectroscopy as presented in Section 3.5.

### 3.2 Electron microscopy investigations

**FIGURE 2:**
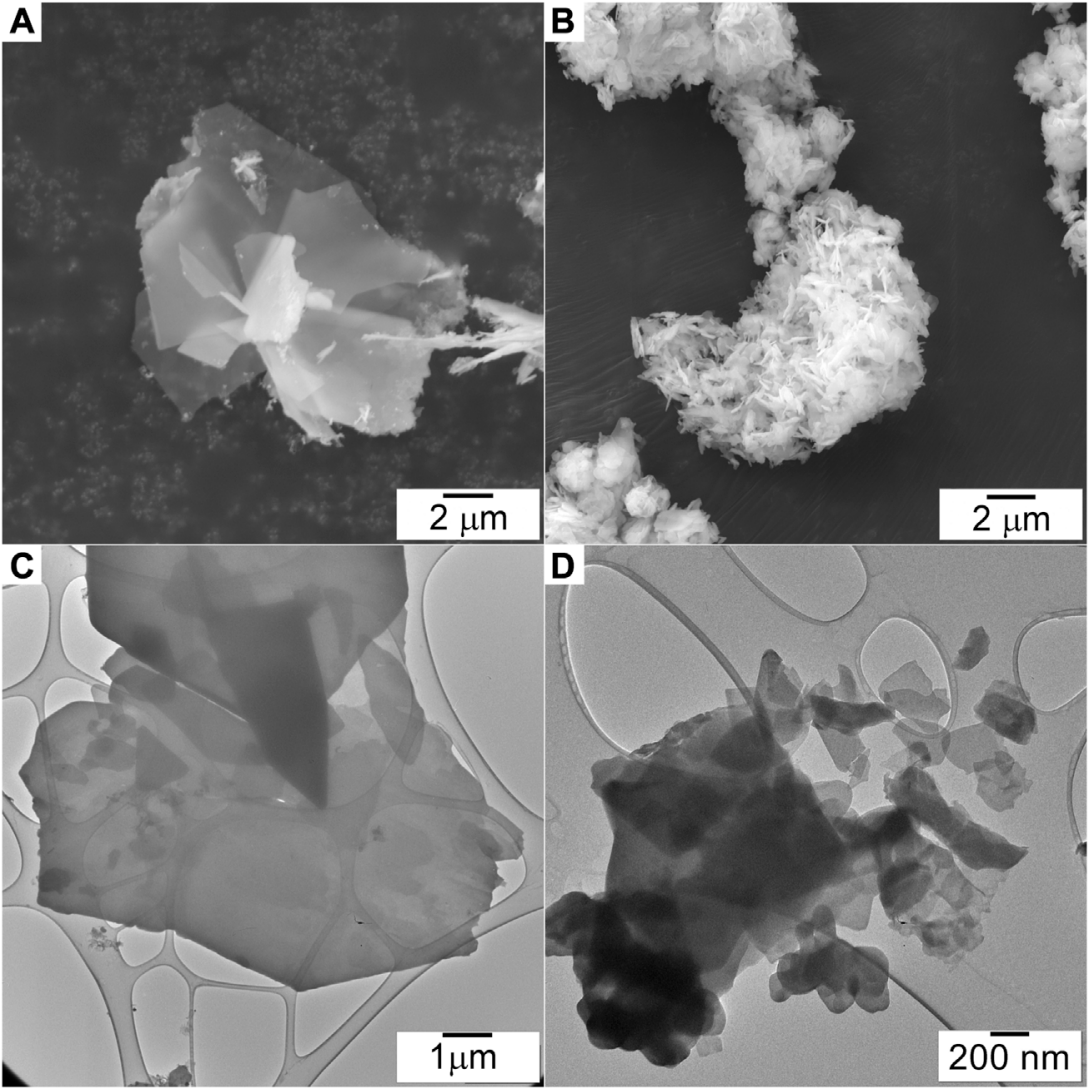
SEM images of the ZHN_COPP_ **(A)** and ZHN_SSR_ **(B)** samples. TEM images of the ZHN_COPP_ **(C)** and ZHN_SSR_ **(D)** samples.

SEM. SEM images of the ZHN_COPP_ and ZHN_SSR_ samples are shown in Figures 2A, B. Both samples consist of agglomerated platelets shaped as irregular polygons, randomly oriented, but with much larger dimensions (up to about 10 μm) for the ZHN_COPP_ sample. Because of this dimensional difference, the two samples observation had to be performed with different microscope magnitudes, as seen in Figure 2. Both samples display the characteristic plate-like morphology of the layered materials, with nanometric-scale thick platelets, thinner than previously reported (Li et al., 2012; de Oliveira and Wypych, 2016; Ruiz et al., 2020). A comparison of Figures 2A, B evidences a more pronounced shape anisotropy in the case of the ZHN_COPP_ nanocrystals, which can indeed explain the observed texture in the XRD pattern of this sample.

TEM. Low magnification TEM images of the ZHN_COPP_ and ZHN_SSR_ samples are shown in Figures 2C, D. The ZHN_COPP_ sample consists of platelets of large dimensions (few μm to above 10 μm) and much smaller thickness, proven by the fact that the carbon membrane on which particles are supported is still visible. The ZHN_SSR_ sample also consists of platelets but of smaller size, in the range of a few hundred nanometers. Both samples were very sensitive to the electron beam, a fact that hindered the analysis at higher magnification, although damage prevention measures were taken (the accelerating voltage was set to 80 kV and the images were taken using low beam intensity and longer exposure times).

### 3.3 Textural analysis

**FIGURE 3:**
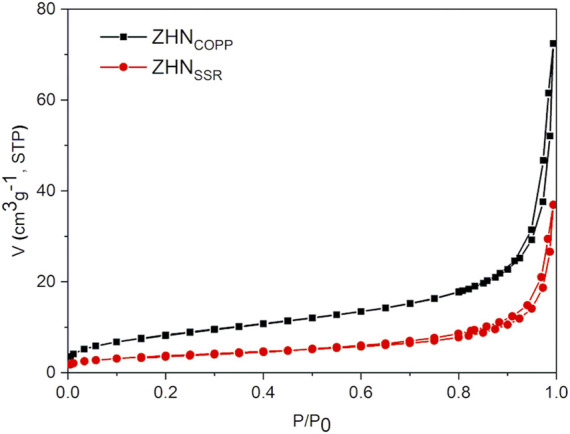
N_2_ adsorption–desorption isotherms of the samples.

The N_2_ adsorption–desorption isotherms of the samples are shown in Figure 3. Both isotherms can be classified as type IV according to IUPAC classification, with small hysteresis loops of H3 type (Sing et al., 1985). Both specific surface area and total pore volume are more than two times larger for the ZHN_COPP_ sample than for the ZHN_SSR_ sample, as seen in Table 1. These values are in agreement with those reported in the literature for materials obtained by similar methods (Reinoso et al., 2014). However, the mean diameter of the pores is smaller for ZHN_COPP_ than for ZHN_SSR_. The notable differences in the specific surface area and total pore volume are correlated with the different size/morphology observed by electron microscopy for the two ZHN nanostructures. The appearance of the hysteresis loop at relative pressure values higher than 0.9 indicates the existence of relatively large pores, most likely coming from the interparticle voids.

**TABLE 1:** Textural characteristics of the two ZHN samples.

Sample	S_BET_ (m^2^g^−1^)	Total pore volume (cm^3^g^−1^)	Average pore size (nm)
ZHN_COPP_	30.3	0.112	14.9
ZHN_SSR_	13.1	0.057	18.8

To our knowledge, the results presented in Sections 3.1–3.3 represent the first comparative study of the morpho-structural properties of the ZHN compounds obtained by two different methods. By using two different synthesis methods, but keeping as many synthesis parameters similar as possible, i.e., the same precursors, Mn doping level, precipitate washing process and drying temperature and time, we have obtained ZHN nanostructures with notably different morphological properties. Up to now there are only a few reports on the morphology of the ZHN compounds (Hussein et al., 2009; Li et al., 2012; Reinoso et al., 2014; de Oliveira and Wypych, 2016; Ruiz et al., 2020), and no correlation was made with the synthesis method. It is generally known that the size and morphology at nanoscale are influenced by several factors, including the synthesis method itself, changes in the synthesis or post-synthesis parameters, doping, etc., and changes in just one parameter could result in a spectacular shape change (Yang et al., 2013; Ghica et al., 2016; Guria and Pradhan, 2016). In our case, it is possible that the mechanical stress due to the grinding and also the lack of any solvent (other than the water adsorbed from the atmosphere) affected the growth of the ZHN_SSR_ platelets, impeding the development of highly anisotropic shapes, while for ZHN_COPP_ the preparation conditions were more suitable for the growth of well crystallized platelets, in the solvent presence. Despite intensive research in this field, growth mechanisms are still a difficult issue even for simple oxide, sulfide, selenide structures (ZnO, ZnS, ZnSe, CdSe, etc.) and further experimental and theoretical research is required (Yang et al., 2013; Guria and Pradhan, 2016).

### 3.4 FTIR results

**FIGURE 4:**
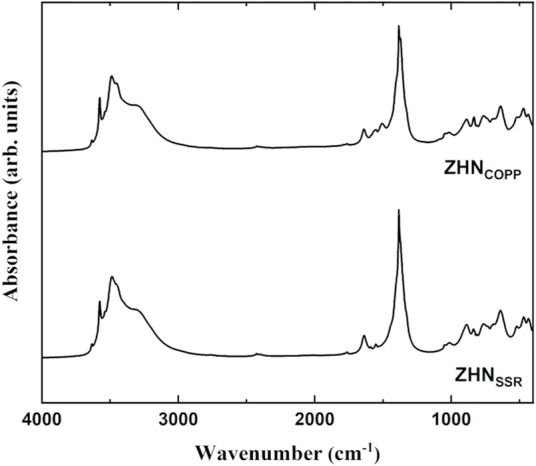
Infrared spectra of the ZHN_COPP_ and ZHN_SSR_ samples.

As it can be observed in Figure 4, the two ZHN samples exhibit the same absorption bands previously reported for the Zn_5_ (OH)_8_ (NO_3_)_2_·2H_2_O compound (Stählin and Oswald, 1971; Chouillet et al., 2004). The sharp peak around 3,570 cm^−1^ and the intense peak at 3,400 cm^−1^ are assigned to various stretching vibrations of the O-H bonds in Zn_5_ (OH)_8_ (NO_3_)_2_·2H_2_O (Chouillet et al., 2004). The broad band around 3,300 cm^−1^ and the peak around 1,640 cm^−1^ indicate the presence of water molecules in the interlayer space or adsorbed on the surface. For both spectra shown in Figure 4, the most intense peak at about 1,380 cm^−1^ corresponds to the *ν*_*3*_ vibration (NO_2_ stretching mode of a free nitrate ion), while the weak peaks around 1,050 cm^−1^ correspond to the *ν*_*1*_ vibration (NO stretch of a free nitrate group), 840 cm^−1^ to the *ν*_*2*_ vibration (out of plane vibration) and 720 cm^−1^ to the *ν*_*4*_ vibration (NO_2_ bend), characteristic to various vibration modes of the nitrate group (Stählin and Oswald, 1971; Chouillet et al., 2004). It should be emphasized that, as observed by Chouillet et al. (2004), the 1,380 cm^−1^ absorption band is not split, in agreement with Zn_5_ (OH)_8_ (NO_3_)_2_·2H_2_O structure in which the nitrate ion is not coordinated (Louër et al., 1973). Moreover, the weak absorption band at 1,050 cm^−1^ is infrared forbidden and should not appear in a *D*_*3h*_ symmetry, but it can be present when the symmetry is slightly distorted (Gatehouse et al., 1957). Below 1,000 cm^−1^ translation and bending vibration modes for Zn-OH, nitrate group and Zn-O bonds, with low intensity, can be observed in the spectra of both compounds (Jnido et al., 2021).

### 3.5 EPR investigation

**FIGURE 5:**
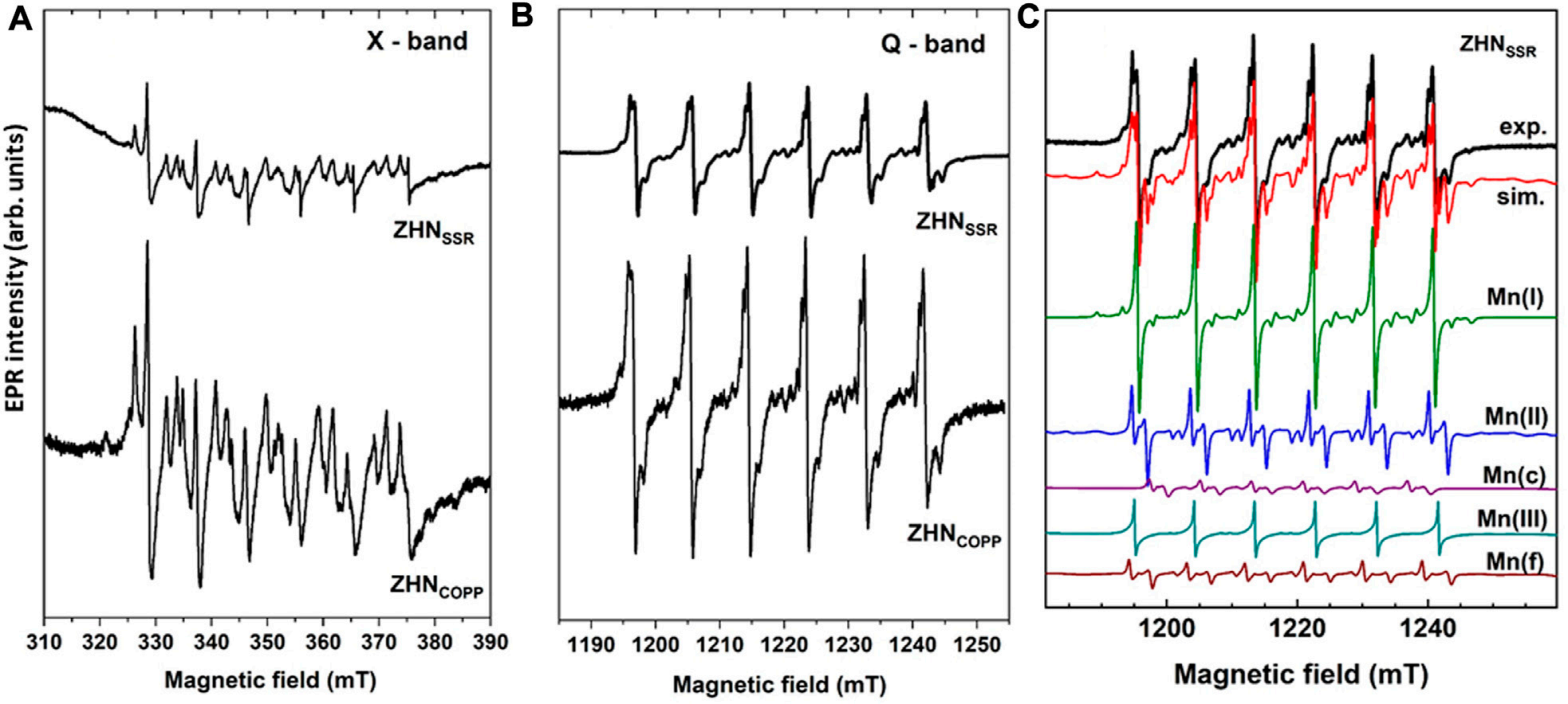
EPR spectra of the as-prepared samples measured at RT in X-band (9.86 GHz) **(A)**, and Q-band (34.12 GHz) **(B)**. **(C)** Experimental (exp.) and simulated (sim.) EPR spectrum in Q-band of the ZHN_SSR_ sample. The component spectra of the indicated centers, calculated with the SH parameters from Table 2, are presented below.

The EPR spectra of the as-prepared samples in the X (9.86 GHz) and Q (34.12 GHz) frequency bands, normalized for differences in mass and spectrometer parameters, are presented in Figures 5A, B, respectively. For each sample the EPR spectrum consists of several overlapping individual spectra with a characteristic six-lines hyperfine structure (better observed in Q-band, Figure 5B), attributed to Mn^2+^ ions (*S* = 5/2, *I* = 5/2) localized in different host lattices. The EPR spectra of the two samples are quite similar, with close line widths values and the spectrum of the ZHN_COPP_ sample about three times more intense than the spectrum of the ZHN_SSR_ sample. It can thus be inferred that the Mn^2+^ ions concentration is three times higher in the ZHN_COPP_ sample than in the ZHN_SSR_ sample, pointing to an enhanced doping efficiency when the coprecipitation synthesis route is used. We associate this result to the enhanced shape anisotropy of the ZHN_COPP_ nanocrystals, in agreement with theoretical studies which show that the doping efficiency varies with the morphology and size of the nanocrystals (Erwin et al., 2005; Singh et al., 2010).

The EPR spectra of the Mn^2+^ paramagnetic centers were analyzed using the following spin Hamiltonian (SH), where the first term is the electron Zeeman interaction of the *S* = 5/2 electron spin with the external magnetic field *B* and the second term is the hyperfine interaction of the electron spin with the *I* = 5/2 nuclear spin of the ^55^Mn (100% abundance) isotope, responsible for the characteristic six-lines hyperfine structure. The next two terms are zero-field-splitting (*ZFS*) terms describing the interaction of the electron spin with the local crystal field, while the last term is the nuclear Zeeman interaction (Abragam and Bleaney, 1970; Stefan et al., 2014):

$$\begin{array}{l} H=\mu_{B}S\cdot g\cdot B+S\cdot A\cdot I+\frac{a}{6}\left[\left(S_{x}^{4}+S_{y}^{4}+S_{z}^{4}\right)-\frac{1}{5}S\left(S+1\right)\left(3S^{2}+3S-1\right)\right]\\ +D\left[S_{z}^{2}-\frac{1}{3}S\left(S+1\right)\right]-\mu_{N}g_{N}B\cdot I \end{array}$$

For nanostructured materials, the contribution of random strains and lattice disorder is very important, producing a variation in the local crystal field at the paramagnetic ion sites, both in magnitude and orientation, reflected in the broadening of the individual EPR line width. This line broadening is reproduced in the lineshape simulation of the experimental spectra by including a statistical distribution of the axial *ZFS*-parameter value *D*, described by the corresponding standard deviation σ(*D*) (Stefan et al., 2013). The EPR parameters (*g, A, D, a*) of the Mn^2+^ centers, σ(*D*) and individual linewidth (Δ*B*), as determined by simulation and fitting of the Q-band spectrum of the ZHN_SSR_ sample are presented in Table 2. The Mn(I) and Mn(II) centers correspond to Mn^2+^ ions in ZHN, while the Mn(III) centers are Mn^2+^ ions localized in a Zn_3_ (OH)_4_ (NO_3_)_2_ phase (Ghica et al., 2019). Mn(c) corresponds to Mn^2+^ ions in crystalline ZnO (Stefan et al., 2013), while Mn(f) is associated to traces of doped precursor. The simulated Q-band spectrum of the ZHN_SSR_ sample with all the above mentioned contributions (Table 2) is presented in Figure 5C. The relative concentrations of the Mn^2+^ centers in the respective hosts, given in Table 2 with an accuracy of ±2%, are proportional to the integrated intensities of the associated spectra. Thus, in the ZHN_SSR_ sample, the concentration of the Mn^2+^ ions in traces of ZnO and precursors is much lower than the concentration of the Mn^2+^ ions in ZHN. On the other hand, the EPR spectrum of the ZHN_COPP_ sample shows that the Mn^2+^ ions are localized substitutionally only in the two Zn^2+^ sites from ZHN.

**TABLE 2:** Relative concentration, EPR parameters (*g, A, D* and *a*), broadening parameter σ (*D*) and individual peak-to-peak linewidth (ΔB) of the Mn^2+^ centers in the ZHN samples.

Sample	Center/relative concentration	*g*	*A* [10^–4^ cm^−1^]	*D* [10^–4^ cm^−1^]	*a* [10^–4^ cm^−1^]	σ (*D*) [%*D*]	Δ*B* [mT]
ZHN_SSR_	Mn(I)/22%	2.0011 ± 0.0002	−84.7 ± 0.2	−18 ± 2	9 ± 2	-	0.6
	Mn(II)/53%	2.0009 ± 0.0002	−85.5 ± 0.5	225 ± 5	0	4 ± 1	0.3
	Mn(c)/5%	2.0012 ± 0.0001	−74.0 ± 0.5	−240 ± 5	-	11 ± 1	0.3
	Mn(III)/3%	2.0011 ± 0.0001	−87.5 ± 0.2	-	-	-	0.15
	Mn(f)/17%	2.0013 ± 0.0001	−84.0 ± 0.5	−270 ± 5	-	5 ± 1	0.3
ZHN_COPP_	Mn(I)/39%	2.0011 ± 0.0002	−84.7 ± 0.2	−18 ± 2	9 ± 2	-	0.65
	Mn(II)/61%	2.0009 ± 0.0002	−85.5 ± 0.5	225 ± 5	0	6 ± 1	0.5

The observation of the Mn^2+^ ions in a crystalline phase of ZnO in the ZHN_SSR_ EPR spectrum, with a relative concentration of 5%, lead us to check for the possible presence of ZnO peaks in the XRD patterns of both samples. This minority phase would not be distinguishable in diffractograms, as the corresponding XRD peaks with very low intensities are hidden under the main peaks of the ZHN phase. The Rietveld refinement performed by including ZnO alongside Zn_5_ (OH)_8_ (NO_3_)_2_·2H_2_O resulted in a better fit of the diffractograms and allowed the identification of traces (1%–2%) of ZnO hexagonal phase (space group P63mc, ICDD 01-070-8070) with average crystallite size of ∼15 nm in both samples.

The spectral parameters of the two Mn-related centers [Mn(I) and Mn(II)] in ZHN are similar in both samples and correspond to the ones reported by Ghica et al. (2019), indicating the presence of the Mn doping ions in the sample volume for both synthesis methods. Along with the structural investigations, this result shows similar structural quality at atomic scale of both ZHN nano-systems obtained by the two synthesis methods, even though ZHN_SSR_ contains traces of precursor phases.

**FIGURE 6:**
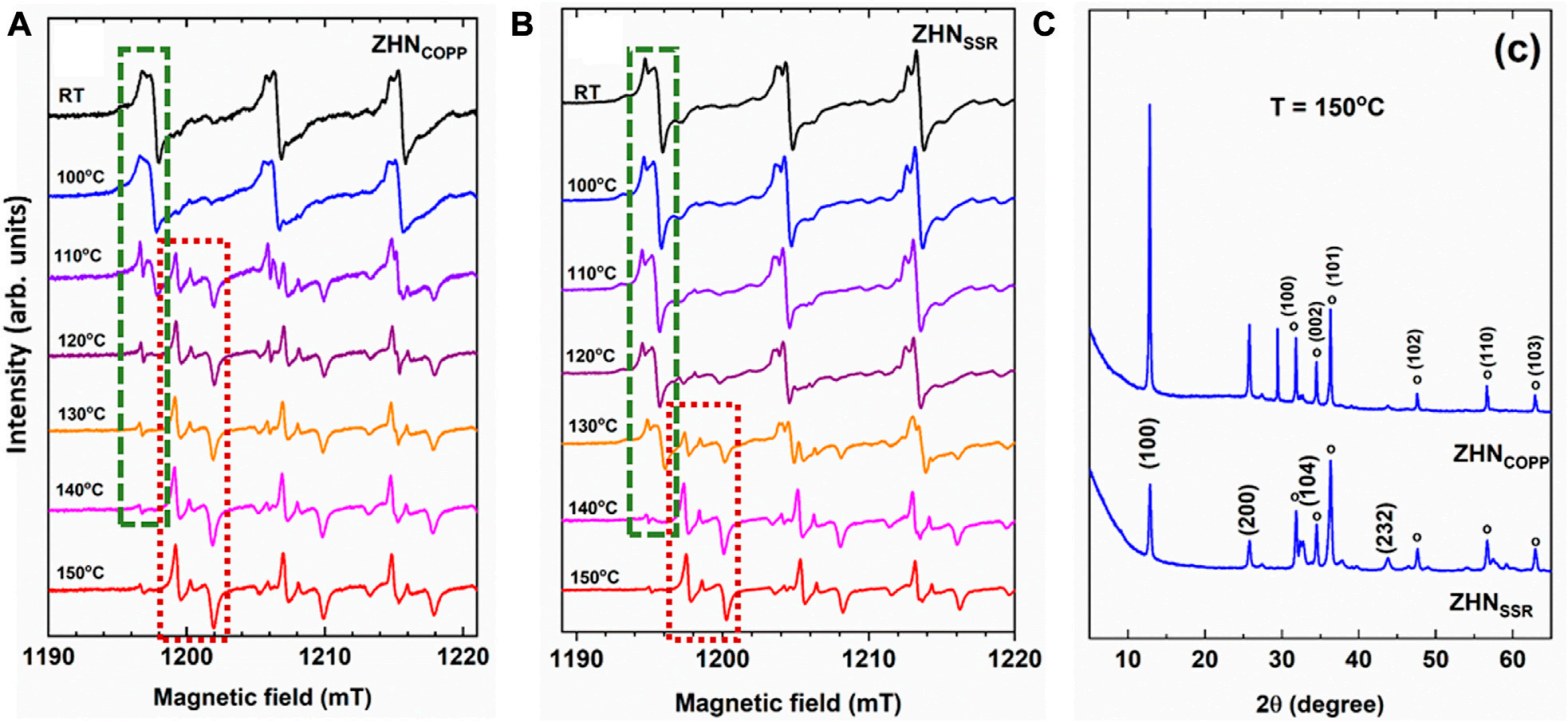
Detailed view of the EPR spectra of the Mn^2+^ ions in the ZHN_COPP_ **(A)**, and ZHN_SSR_ **(B)** samples, as-prepared (RT) and isochronally annealed (10 min at each indicated temperature). Only the first three hyperfine transitions at low-field are displayed for simplicity. Transformation of the Mn^2+^ centers in the zinc hydroxynitrate phases into Mn^2+^ centers in ZnO is evidenced with green and dark red dotted lines, respectively, only for the first hyperfine transition, where these centers do not overlap. **(C)** XRD patterns of both samples after isochronal annealing up to 150°C. The ZnO phase is marked with open circles and indexed in the upper pattern, while the Zn_3_ (OH)_4_ (NO_3_)_2_ phase is indexed in the lower pattern.

The thermal decomposition of both ZHN samples has been investigated by EPR using the Mn doping ions as paramagnetic probes. Their EPR spectra after isochronal annealing experiments up to 150°C are represented in Figures 6A, B, where only the first three hyperfine transitions at low-field are displayed for simplicity. At temperatures above 100°C the Mn(I) and Mn(II) EPR spectra become weaker, and new centers are formed. The thermal evolution of the Mn^2+^ centers reflects the evolution of their host material, namely, the decomposition of ZHN and the formation of new phases. A notable increase in the intensity of the EPR spectrum of Mn(c) (Mn^2+^ ions in ZnO nanocrystals) appears at 110°C for the ZHN_COPP_ sample, and at 130°C for the ZHN_SSR_ sample. We should mention that a similar EPR investigation of the thermal decomposition of crystalline Zn(OH)_2_ showed the on-set of the formation of nanocrystallline ZnO at 120°C (Nistor et al., 2013b). We note that in the ZHN_COPP_ sample, the Mn(III) centers are formed only above 100°C. For both samples the Mn(III) centers decrease in intensity with the annealing temperature increase, being hardly visible at 150°C (Figures 6A, B). For an easier observation of the evolution of the Mn^2+^ spectra from Figure 6A, B we have marked the first hyperfine transition at low-field of the Mn^2+^ centers with a green rectangle for all zinc hydroxynitrate compounds [Mn(I) and Mn(II) in Zn_5_ (OH)_8_ (NO_3_)_2_·2H_2_O and Mn(III) in Zn_3_ (OH)_4_ (NO_3_)_2_], and with a dark red rectangle for ZnO.

After isochronal annealing up to 150°C, the EPR spectra of both samples show mostly the Mn(c) paramagnetic center, meaning that almost all Mn^2+^ ions are localized in the volume of the ZnO phase. However, the corresponding XRD patterns of both samples consist of a mixture of two different crystalline phases: Zn_3_ (OH)_4_ (NO_3_)_2_ and ZnO (Figure 6C), with an enhanced transformation towards ZnO in the case of the ZHN_SSR_ sample. It can thus be concluded that the transformation of ZHN into ZnO is initiated at lower temperatures in the sample regions containing manganese than in the pure ones, independent of the synthesis routes, as further explained. The doping process can be used to change or modulate the morpho-structural properties of nanostructured materials, as the doping ions could influence the nanocrystals growth, affecting both the size and morphology, and even induce structural phase transformations or decomposition toward different compounds (Erwin et al., 2005; Ghica et al., 2016; Guria and Pradhan, 2016; Pradhan, 2019). In a previous report, Ghica et al. (2016) have shown that in the case of the Zn (OH)_2_ compound, the minute addition of Mn^2+^ ions (1 ppm) re-directed the coprecipitation synthesis toward Mn-doped ZnO instead of Mn-doped Zn (OH)_2_. It was suggested that this effect could be driven by a coordinative hindrance suffered by Mn^2+^, which in most compounds assumes an octahedral coordination, when substituting for Zn^2+^ in a site tetrahedrally coordinated by four (OH)^−^ ligands. As mentioned in Section 3.1, there are two cationic sites in the ZHN compound: One with Zn^2+^ ions octahedrally coordinated by six (OH)^−^ groups and one with Zn^2+^ ions tetrahedrally coordinated by three (OH)^−^ groups and one water molecule. It is possible that the Mn^2+^ ions substituting for Zn^2+^ ions in tetrahedral sites could favor the transformation into ZnO by a similar mechanism. The tetrahedrally coordinated sites could thus become nucleation sites for ZnO at a lower temperature than in the undoped material. In our previous study of ZnO nanostructures obtained by coprecipitation followed by annealing (Ghica et al., 2019), we have observed that the transformation of ZHN into ZnO was initiated at lower temperatures in the sample regions containing manganese than in the pure ones. As shown in the present work, the ZHN to ZnO transformation at a lower temperature at the Mn^2+^ sites takes place for both ZHN compounds with different morphologies obtained by two different synthesis methods. This effect is thus the result of the doping process itself, and not dependent on the synthesis method.

On the other hand, the difference in the onset temperature for the phase transformation in the two nanostructured ZHN samples (110°C for ZHN_COPP_ and 130°C for ZHN_SSR_) is related to their different size and morphology resulted from the specific synthesis method used. This correlation has been detailed in the last paragraph of Section 3.3, following the morpho-structural analysis. Although both ZHN samples have the same nominal doping level, the EPR study has shown that the concentration of the substitutional Mn^2+^ ions is higher in the ZHN_COPP_ sample, therefore providing more nucleation sites for ZnO in the ZHN_COPP_ sample than in the ZHN_SSR_ sample. This result can also explain the lower onset temperature for the transformation into ZnO in the ZHN_COPP_ sample than in the ZHN_SSR_ sample.

Our result points to a more convenient lower temperature synthesis of ZnO nanocrystals by thermal decomposition of ZHN obtained either by coprecipitation or solid state reaction, when ZHN is doped with Mn^2+^ ions. This subject deserves further investigation, towards the identification of new cost-effective synthesis routes of Mn^2+^ doped nano-ZnO. Doping in general and Mn^2+^ doping specifically in the case of nanostructured ZnO can modulate/enhance material properties for different applications (Sharma et al., 2003; Kittilstved and Gamelin, 2006; Dietl, 2010; Hoye et al., 2013; Jiang et al., 2018; Martínez-Carmona et al., 2018; Zhang et al., 2018; Pradhan, 2019; Popescu et al., 2020; Pradeep and Viswanatha, 2020) as a function of the dopant concentration and localization. However, the synthesis of nanostructured doped ZnO is not trivial, as shown by the still not-resolved issues regarding the control and uniformity of the dopant distribution and solubility, agglomeration and formation of minority phases, all depending on the synthesis method and procedures. Thermal decomposition at low temperatures of Mn^2+^ doped ZHN promises to be a good method to obtain uniformly doped ZnO nanocrystals.

To summarize, a comparison of the two synthesis routes for ZHN seems to incline the balance in favor of the coprecipitation route. The two samples have similar structure and crystallite size, the dopant Mn^2+^ ions are easily incorporated in the host lattices, but the coprecipitation sample has a higher doping efficiency. However, the solid state reaction method has the non-negligible advantage of a cleaner and simpler process, with less reactants involved, and a higher thermal stability of the as-prepared ZHN sample.

## 4 Conclusion

Nanocrystalline Zn_5_ (OH)_8_ (NO_3_)_2_·2H_2_O (ZHN), doped with a small amount of Mn^2+^ ions, was prepared by two different synthesis routes, coprecipitation and solid state reaction using the same environment-friendly reactants. The resulting nanocrystalline samples have distinct morpho-structural and textural properties, clearly influenced by the synthesis method. While in both cases the structure and crystallite size are similar, the sample obtained by coprecipitation exhibits much larger platelets with specific surface area and total pore volume more than two times larger than in the case of the sample obtained by solid state reaction. From the analysis of the EPR spectra we determined that the Mn^2+^ dopant ions are localized substitutionally in the Zn^2+^ sites of the host lattice in both samples. The concentration of the Mn^2+^ ions is three times higher in the coprecipitation sample, showing that this synthesis route ensures an enhanced doping efficiency. The on-set of the ZHN structural phase transformation toward ZnO was observed by EPR at 110°C and 130°C for the samples synthesized by coprecipitation and solid state reaction, respectively. A comparison of the EPR results with the structural and compositional XRD results showed that the Mn^2+^ dopant ions promote the local transformation of ZHN into ZnO at lower temperatures. This is the first detailed investigation of the decrease in the ZHN to ZnO transformation temperature initiated by the Mn^2+^ doping.

Our results provide a basis for the selection of the appropriate synthesis method for nanocrystalline ZHN suitable for the envisaged application in nanotechnology, healthcare or agriculture and for the development of new green, cost-effective synthesis routes of Mn^2+^ doped nano-ZnO.

## Data Availability

The original contributions presented in the study are included in the article/supplementary material, further inquiries can be directed to the corresponding author.
